# Sensory synergy as environmental input integration

**DOI:** 10.3389/fnins.2014.00436

**Published:** 2015-01-13

**Authors:** Fady Alnajjar, Matti Itkonen, Vincent Berenz, Maxime Tournier, Chikara Nagai, Shingo Shimoda

**Affiliations:** Intelligent Behavior Control Unit, Brain Science Institute-TOYOTA Collaboration Center of RIKENNagoya, Japan

**Keywords:** prosthetic arms, sensorineural feedback, muscle synergy, sensory synergy, posture control, automatic posture response

## Abstract

The development of a method to feed proper environmental inputs back to the central nervous system (CNS) remains one of the challenges in achieving natural movement when part of the body is replaced with an artificial device. Muscle synergies are widely accepted as a biologically plausible interpretation of the neural dynamics between the CNS and the muscular system. Yet the sensorineural dynamics of environmental feedback to the CNS has not been investigated in detail. In this study, we address this issue by exploring the concept of sensory synergy. In contrast to muscle synergy, we hypothesize that sensory synergy plays an essential role in integrating the overall environmental inputs to provide low-dimensional information to the CNS. We assume that sensor synergy and muscle synergy communicate using these low-dimensional signals. To examine our hypothesis, we conducted posture control experiments involving lateral disturbance with nine healthy participants. Proprioceptive information represented by the changes on muscle lengths were estimated by using the musculoskeletal model analysis software SIMM. Changes on muscles lengths were then used to compute sensory synergies. The experimental results indicate that the environmental inputs were translated into the two dimensional signals and used to move the upper limb to the desired position immediately after the lateral disturbance. Participants who showed high skill in posture control were found to be likely to have a strong correlation between sensory and muscle signaling as well as high coordination between the utilized sensory synergies. These results suggest the importance of integrating environmental inputs into suitable low-dimensional signals before providing them to the CNS. This mechanism should be essential when designing the prosthesis' sensory system to make the controller simpler.

## Introduction

Neuroprosthetics faces considerable challenges, especially when it is necessary to account for neurological disorders (Ring and Rosenthal, 2005). These challenges concern mainly the immense variety of possible neural damage, which make it hard to define a reliable sensorimotor pathway for controlling external devices (Musallam et al., 2004). Conventional prosthetics focuses mainly on motor control and pays less attention to the role of integrating sensory information as feedback. Without sensory feedback, even the simplest actions, such as controlling a prosthetic arm, can be slow and clumsy due to the lack of tactile sense (Kwok, 2013). Some researchers have proposed direct sensory feedback through air pressure or electrical stimulation though these methods have a number of limitations. Neurophysiological studies have found that the body position in space is estimated by integrating information from multiple sensors modalities rather than through direct sensory input (Zupan et al., 2002; Mergner et al., 2003; Kuo, 2005; Ting, 2007). This integrated sensory feedback can encode noise-robust, useful, and cost-effective information in low-dimensional signals that are simple enough to accelerate the construction of the desired control signal (Kargo and Giszter, 2000). Adding proper sensory integration mechanism into the design of the prosthesis, therefore, may propose an access to simpler controller.

In recent years, several studies have indicated that muscle synergy is a likely neural strategy that the central nervous system (CNS) has adopted to simplify the control of our redundant musculoskeletal system (D'Avella and Bizzi, 2005; Safavynia et al., 2011; Alnajjar et al., 2013b). The concept of muscle synergy, therefore, has been widely adopted as a quantitative interpretation of motor control strategies on a neural level. Muscle synergy has been investigated in detail in several areas of research, including the clarification of the corresponding anatomical concept (Bizzi and Cheung, 2013), the classification of the motor skills of healthy subjects (Torres-Oviedo and Ting, 2007; Alnajjar et al., 2013a), the synergetic motor control paradigm for managing joint redundancy (Hayashibe and Shimoda, 2014) and the identification of the degree of brain damage in stroke survivors (Cheung et al., 2012).

**Figure 1:**
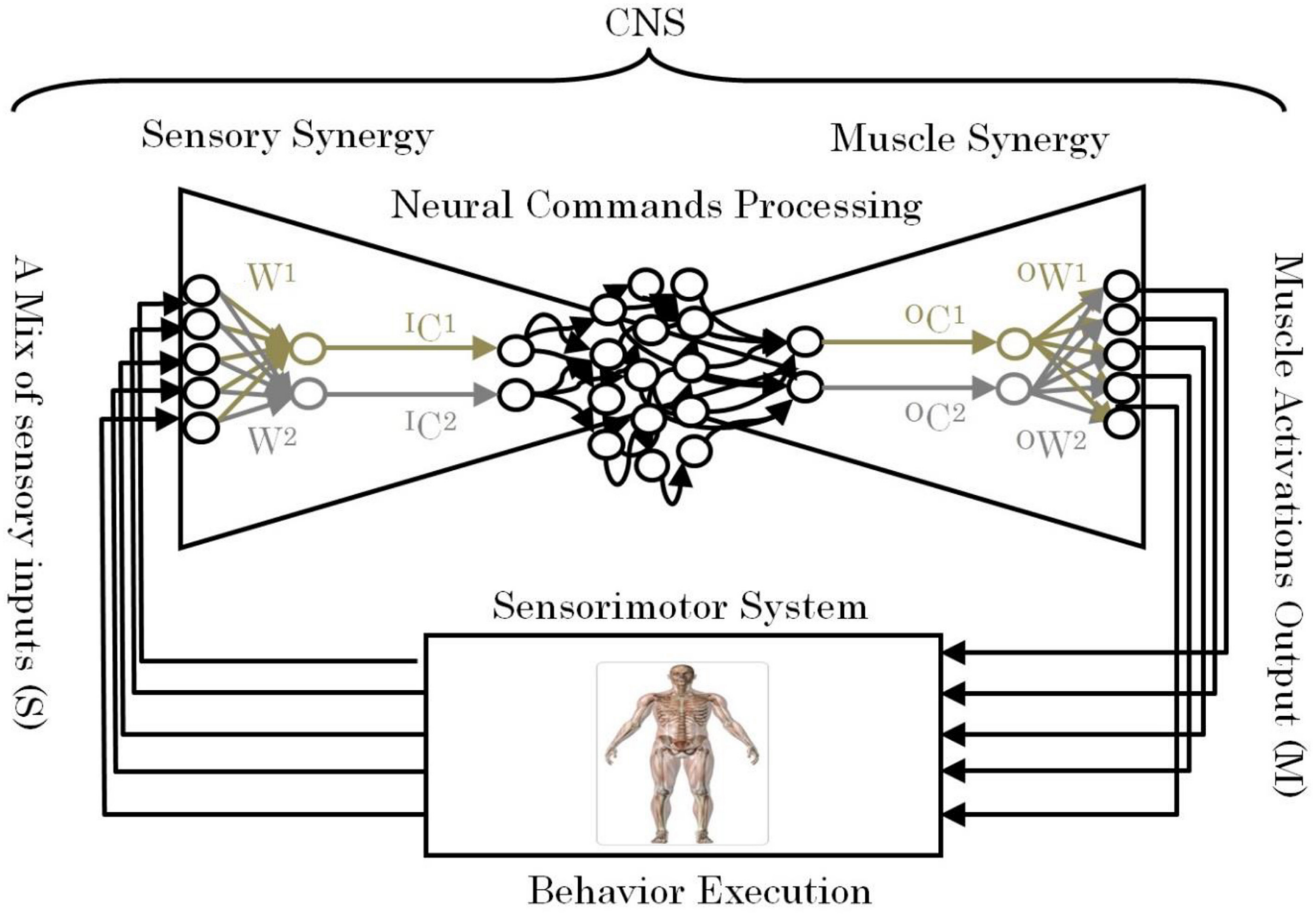
**Conceptual model of a neural sensorimotor synergy system**. An example of two input sensory synergies (*W^(1),(2)^*, *^I^C^(1),(2)^*) and two output muscle synergies (*^O^W^(1),(2)^*, *^O^C^(1),(2)^*).

One of the remaining unsettled debates concerning muscle synergies is how they are selected and evaluated by the CNS to adapt to the surrounding environment including body dynamics (Latash, 2008). Answering this fundamental question is essential to understanding the mechanism used by the CNS to handle the complexity of sensorimotor interactions in the body. To answer this question, we introduce the term “sensory synergy” to supplement muscle synergy in order to understand the mechanism of mapping stimuli to behavior (Figure 1). In contrast to muscle synergy, which defines suitable combinations of muscles to adapt the behavior to the environment, we hypothesize that sensory synergy plays essential roles in integrating a compendium of sensory feedback to simplify the construction of muscle synergy. We define a single sensory synergy as a group of weighted sensory inputs whose function is to provide the quality of the resulting motion as feedback to the CNS through a single synergy recruitment signal in order to facilitate the generation of the next command, thus accelerating the search time for the optimal muscle synergy. Sensory synergies studies could be the simplified way to understand sensory signaling. In nature, sensory signals of different modalities are in general redundant and plastic to ensure delivering appropriate environmental information to the CNS (Day and Guerraz, 2007). If one sensory modality is disrupted or become unavailable, the other modality can take over (Dickstein et al., 2001; Lanska, 2002). In some cases one of the sensory modalities can even override all others modalities and drives them (Diedrichsen et al., 2007).

To conduct this study, we recorded the kinematics patterns and muscle activities of nine healthy participants in an automatic posture response experiment (APR). The results highlight the synergy characteristics common to all individuals, which were found to depend on the quality of their APRs skills. Results revealed a potential link between the sensory and muscle synergies in terms of synergy size that may enhance sensorimotor transformations. This study should be useful to inspire the development of sensory system for effective neural prosthetic devices which can be operated with simple controller.

## Materials and methods

### Experimental setup

In this study, an experiment was conducted to determine the relation between the APR measured skills of the participants and their computed sensory and muscle synergies. The participants were nine healthy men (mean age, 34.5 ± 9 years). All the participants were right-handed and had no history of major neurological disorders or posture balance impairment. All experimental protocols were approved by the RIKEN ethics committee.

**Figure 2:**
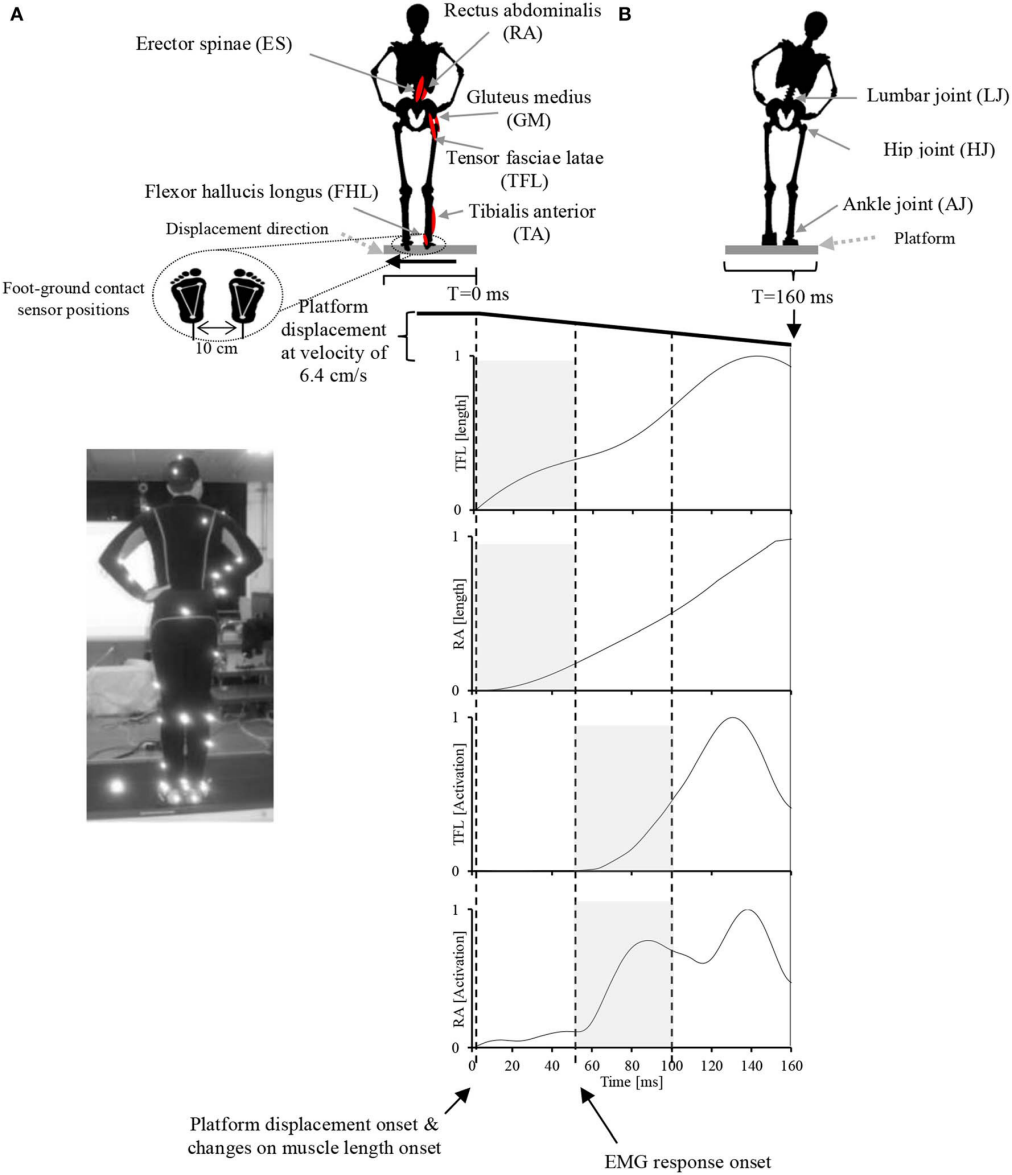
**Experimental setup**. **(A)** A participant in the akimbo position standing on the movable platform. Body motion was captured with a VICON motion capture system using 42 markers attached to various parts of the participant's body (see Supplementary Figure 1 for more information about markers positions). **(B)** Experimental setup, muscle locations, joint locations, platform motion pattern and displacement speed, EMG record range, and representative EMG activities and muscle lengths of two muscles in response to the platform displacement. The participant's EMG responses occurred with a latency of approximately 50 ms following the displacement. The sensory synergy was computed in the period between 0 and 50 ms (shaded area in the muscle length plots), and muscle synergy was computed in the period between 50 and 100 ms (shaded area in the muscle activation plots).

During the experiment, the participants were instructed to stand upright in the akimbo position (Figure 2A) on a movable platform, placing their feet on foot-ground contact sensors located approximately 10 cm apart (Figure 2B). We chose this standing position, in which hands are placed on a little above the hips and the elbows are bowed outward, to reduce any impact of the arms in restoring the body balance and to facilitate the capturing of motion markers attached on the participants' bodies. The platform was programmed to perform lateral displacements of 11 cm with velocity of 6.4 cm/s. The participants were also instructed to make an effort to maintain their balance in an upright posture during the platform displacements and to avoid any body movements other than lateral hip flexion/extension and ankle inversion/eversion. The direction and timing of displacement was chosen at random and therefore it was unpredictable to the participants during the experiment. Before the experiment, each participant was asked to practice balancing on the platform for 20 min to become familiar with the experimental environment. At the time of the experiment, each participant experienced leftward and rightward platform displacements (mean ± SD: 18 ± 4 cm), and electromyograms obtained in five trials of leftward displacement were used for data analysis.

### Datas recording

#### Surface electromyography (EMG)

Data on muscle activity was collected by wireless surface EMG (BTS FREEEMG 300, BTS Bioengineering, Italy). EMG electrodes were used to record data from six dominant leg and lumbar muscles (Hoy et al., 1990): the flexor hallucis longus (FHL) and tibialis anterior (TA), which mainly control the ankle strategy in lateral perturbation; and the tensor fasciae latae (TFL), gluteus medius (GM), rectus abdominalis (RA), and erector spinae (ES), which control the hip strategy and the lumbar joint in lateral perturbation (Runge et al., 1999). The EMG electrodes were placed in accordance to the guidelines of the Surface Electromyography for the Non-Invasive Assessment of Muscles (SENIAM) European Union project (Hermens et al., 1999). The entire time-series EMG data were rectified and processed using a low-pass filter with a cutoff frequency of 32 Hz. EMGs were normalized by their respective maxima measured during the experiment. All signals were resampled to 1 kHz.

#### Motion capture system

Kinematic patterns of the participants' movements were captured with a motion capture system (Workstation 5.2.4, *VICON*). Forty-two markers (spheres covered with reflective tape) were attached to various parts of the participant's body prior to the experiment (see Supplementary Figure 1 for more information about markers positions). The motion capture system consisted of six cameras, and tracked and reconstructed the motion of each of the recorded markers in 3D space.

#### Foot-ground contact sensors

The ground reaction forces for each participant were calculated based on data obtained from foot-ground contact sensors (FingerTPS, Pressure Profile Systems, Los Angeles, CA) distributed over three segments of each foot.

### Estimation of the changes in muscle length

**Figure 3:**
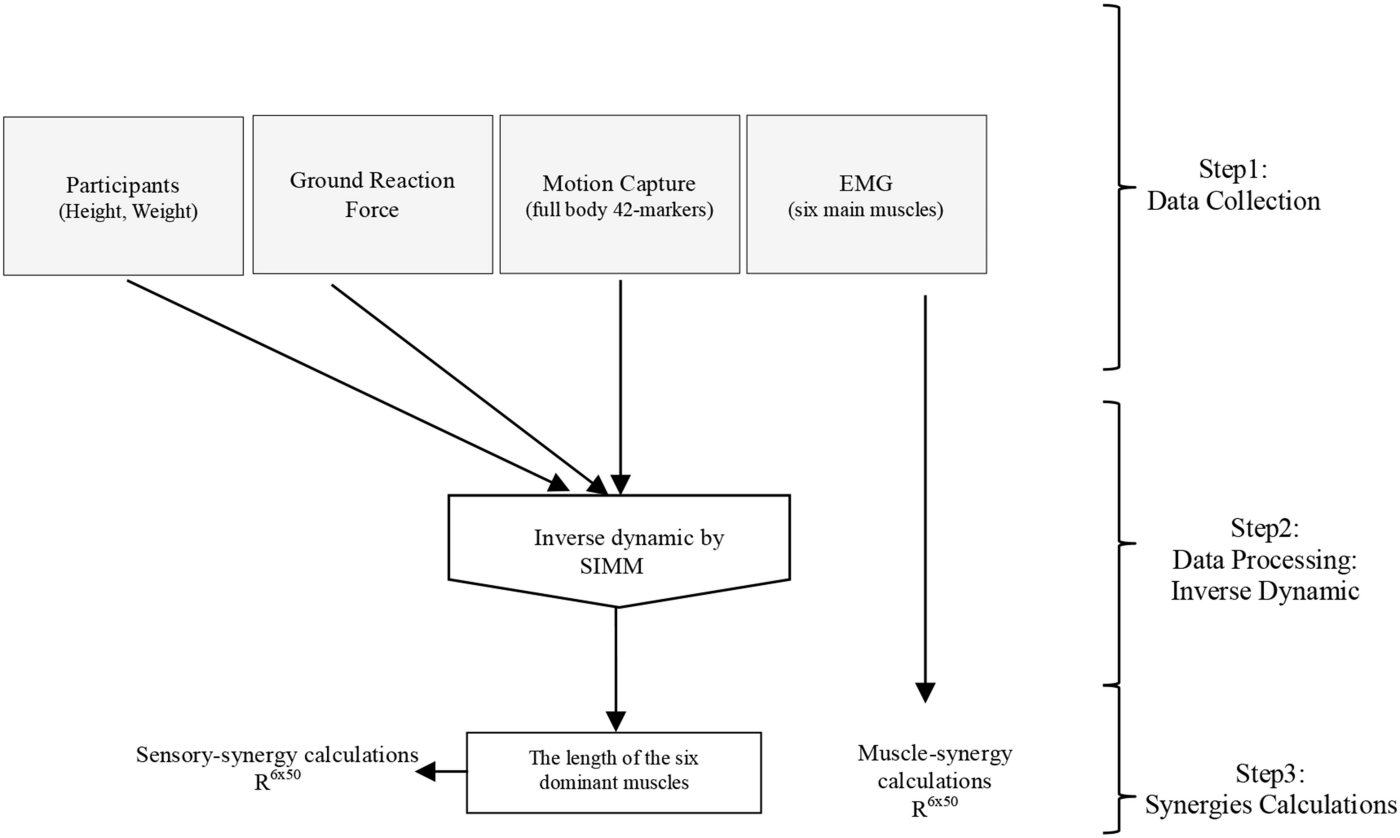
**General procedure for calculating sensory and muscle synergies from the collected experimental data using inverse dynamics calculations in SIMM**.

Software for Interactive Musculoskeletal Modeling (SIMM), a graphical software system for developing and analyzing models of musculoskeletal structures, was used in this study (Delp and Loan, 1995; Neptune et al., 2008). SIMM uses a full body model created by a set of bones from a male adult subject. Muscle parameters in the middle trunk and the lower limb were adjustable according to the scaling bone computed by the recorded markers from the subjects. Each participant's body weight was used to allocate the body segments of the model (de Leva, 1996). SIMM was then used to perform inverse dynamics calculations driven by various data collected from the experiments (i.e., motion capture data, and foot-ground contact sensor data), see Figure 3. Changes in muscle length that is a positive muscle stretch from resting value, as a representation of the activation of proprioceptors (muscle spindles), obtained through inverse dynamics calculations was used as sensory data to compute the sensory synergies. Although 92 muscles and 34 degrees of freedom were considered in the inverse dynamics calculations, due to the simplicity of the applied task (i.e., the fact that the lateral disturbance of a body standing upright can be simplified as a three-link inverted pendulum model (Jiang et al., 2002), and the selected quick and short time period to monitor both sensory and muscle data), we considered sensory synergy calculations using the lengths of the six dominant muscles for which EMG data were recorded are fair enough at this stage (Figure 3). Although it has been argued that the structure of synergies is dependent upon the number and choice of muscles included within the synergies analysis (Steele et al., 2013), we assume that the selected dominant muscles can cope with this issue since they have more influence in carrying out the concerned motion (Alnajjar et al., 2013b; Wojtara et al., 2014). Adding other muscles for synergy calculations should not affect significantly the results (Steele et al., 2013), see also the Supplementary Figure 2.

### Computing sensory synergies

The core feature of sensory synergies is the reduction of the dimensionality of sensory signals provided as feedback to the CNS. Let us express the sensory data of *s* sensors by using a matrix *S*:

(1) $$S\in R^{s\times t},$$

where *s* and *t* are the number of sensors and the sampling number, respectively. The output of the sensor synergy computation *^I^C* is described as follows:

(2) $${}^{I}C=WS$$

where

(3) $${}^{I}C\in R^{n\times t},W\in R^{n\times s}$$

We consider that the sensor synergy computation adds the meaning to the specified combination of the sensor input. The meaningful signal *^I^C* is translated into the muscle synergy input *^O^C* in the low-dimensional space. This signal transfer from *^I^C* to *^O^C* can be considered as the semantic compression of the body-environment interaction in the real environment descried as the M-S transition. *^I^C* can be uniquely estimated from *S* when the well-sophisticated sensor and muscle synergies are used to control the body. Therefore, we assume that the following equation is optimized as the inverse of the muscle synergy computation:

(4) $$S{=}^{I}W^{I}C+E,$$

Where

(5) $${}^{I}W\in R^{s\times n},E\in R^{s\times t}$$

*W* can be regarded as the pseudo-inverse of *^I^W*. We consider that *W* is uniquely computed from *^I^W* when the motion is well-sophisticated. Figure 1 describes the relationships of *S*, *W*, and *^I^C*.

In Equation (4), *n* signals are used to represent *s* sensors by using the sensory synergy *^I^W* and the synergy recruitment *^I^C*. To reduce the dimensionality of the sensory data, we set *n* to be smaller than *s*. The error between *S* and *^I^W ^I^C* is expressed as ***E***, which must be small enough to represent *s* sensors. The magnitude of ***E*** can be described by an index of similarity *L* (Equation 6), which is sensitive to both the shape and the magnitude of the measured and reconstructed sensory patterns (Torres-Oviedo and Ting, 2007):

(6) $$L=100\left(1-\frac{1}{s}\sum_{i=1}^{s}\frac{\sqrt{\frac{1}{t}\sum_{j=1}^{t}E_{ij}^{2}}}{\sqrt{\frac{1}{t}\sum_{j=1}^{t}{S^{\prime }}_{ij}^{2}}}\right),$$

where *S*′ = *^I^*W^*I*^C, and *E_ij_* and *S*′_*ij*_ are the elements of matrices *E* and *S'*, respectively. The range of *L* is 0 < *L* < 100. When the magnitude of ***E*** decreases, *L* increases. We considered a value of *L* > 75% to indicate a good fit to the original data. Through preliminary trial runs, we found that this criterion ensured that each muscle would be reconstructed well. A reasonable value for *n* was chosen by using the index *L* with the non-negative matrix factorization algorithm (NMF) (Lee and Seung, 2001). See **Figure 5** for an example.

### Computing muscle synergy

Muscle synergy was calculated following similar steps as for sensory synergy. The number of signals for representing *m* muscles can be reduced by applying the NMF algorithm using the following matrix:

(7) $$M={\;}^{O}W^{O}C+E,$$

Where in this case

$${}^{O}W\in R^{m\times n}{,}^{O}C\in R^{n\times t},E\in R^{m\times t}$$

Again, *n* signals are used to represent *m* muscles by using the muscle synergy *^O^W* and the synergy recruitment *^O^C*. To reduce the dimensionality of muscle data, we set *n* to be smaller than *m*.

### Sensory synergy size

**Figure 4:**
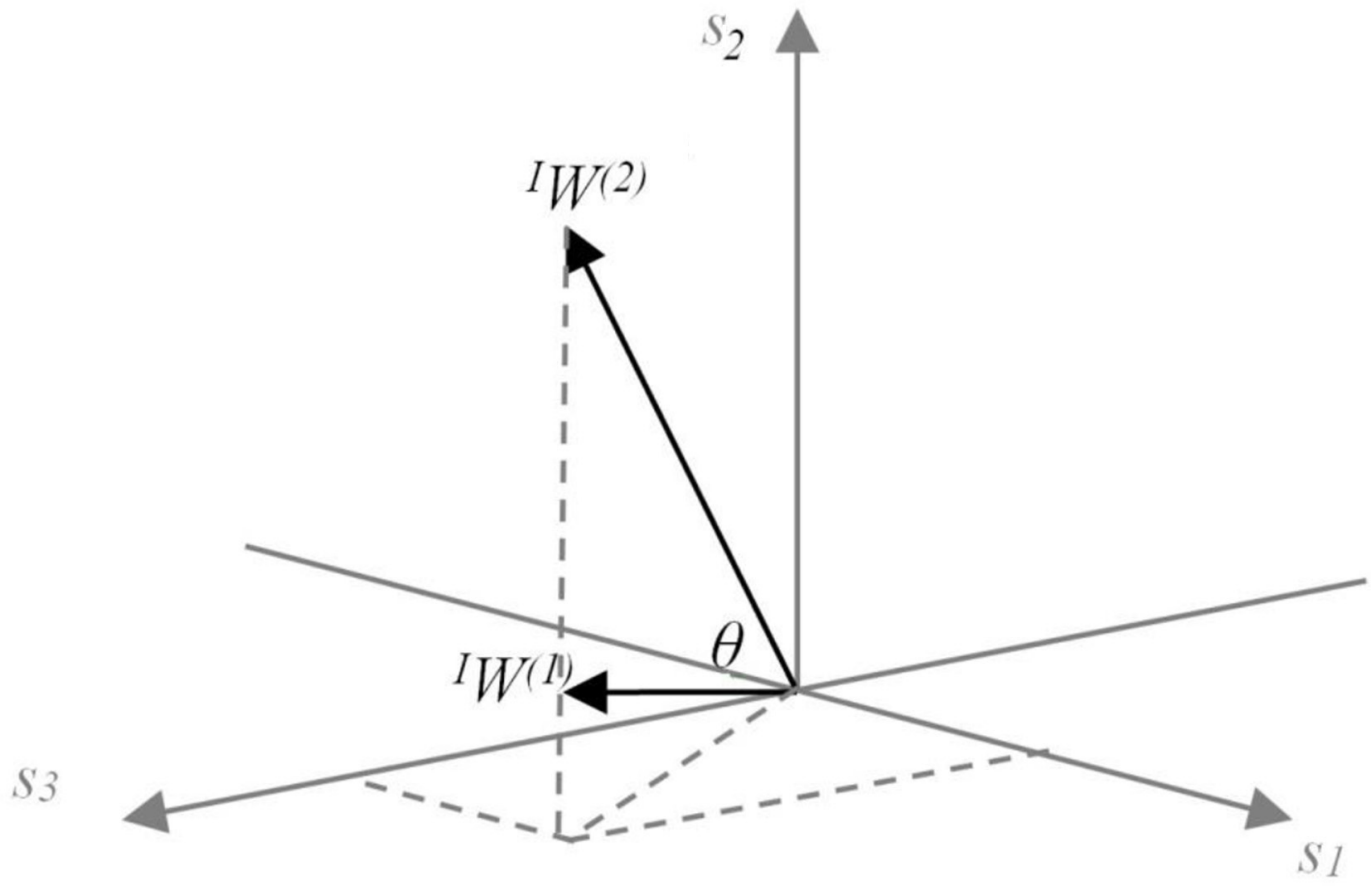
**Conceptual model of the meaning of sensory synergy space size**. Two synergies *^I^W^(1)^* and *^I^W^(2)^* abstracted from three sensory activations s_1_, s_2_, and s_3_, which are positivite. The area of the region between *^I^W^(1)^* and *^I^W^(2)^* represents the size of the synergy space. A similar concept is also applied to the muscle synergy space size by replacing sensor s with muscle m.

The synergy coordination index (SCI) was used to evaluate the resulting synergy space. The space here is represented by the angle θ between the utilized synergies (Figure 4). Let us assume that sensory synergy W is expressed as

(8) $$W=\left[W^{\left(1\right)}\;W^{\left(2\right)}\;W^{\left(3\right)}\;\cdots \;W^{\left(n\right)}\right],$$

where *W*^(*i*)^ ϵ *R*^s^ is a basis vector of the synergy space. Because we use NMF to estimate *W*, the synergy space exists only for positive vector components. Furthermore, vectors *W*^(*i*)^ (*i* = 1 ··· *n*) are in general not orthogonal to each other. The size of the synergy space depends on the relative angles between the vectors *W*^(*i*)^. To quantify the size of the synergy space, we define the space size as the sum of the inner products of *W*^(*i*)^ and *W*^(*j*)^:

(9) $$SCI=\frac{2}{n(n-1)}\sum_{i\neq j}^{n}W^{\left(i\right)}W^{\left(j\right)}.$$

The range of *SCI* is from 0 to 1. *SCI* = 1 implies that all vectors *W*^(*i*)^ are identical, whereas *SCI* = 0 implies that all vectors *W*^(*i*)^ are orthogonal to each other. The synergy space is smaller for larger values of *SCI*.

### Similarity between the sensory/muscle synergy recruitments (S/M similarity)

The S/M similarity describes the similarity between the sensory and muscle synergy recruitment signals *^I^C* and *^O^C* (Figure 1). The S/M similarity is calculated using correlation coefficient:

(10) $$r\left(x,y\right)=|\frac{\sum_{i=1}^{m}\left(x_{i}-\bar{x}\right)(y_{i}-\bar{y})}{mS_{x}S_{y}}|,$$

Here, *x* and *y* are two vectors to be compared (in this case, *^I^C* for *x* and *^O^C* for *y*), *x* and *y* are their mean values, and *S_x_* and *S_y_* are their standard deviations. The S/M similarity ranges from 0 to 1.

A high similarity value indicates that muscle synergy recruitment is highly correlated with sensory synergy recruitment. To avoid the ordering issue in the NMF algorithm, we re-sorted the resulting synergies to obtain the highest similarity.

### Measuring the APR's skill of the participants

To quantify the APR's skill of each participant, a numerical scoring system, based on visual observation by an examiner, was developed (Table 1). To encourage the participants to perform at their best and to maintain a high level of motivation, the scores were also displayed to the participants throughout the experiment on a screen. To ensure the effectiveness and reliability of the scoring system, a video was recorded for all the experiments and the examiner used it to re-score offline the participant performance and compare it to the original scores. Similarity ratios were higher than 98% for all experiments (see an example, Supplementary Video 1). The scoring system was designed to measure the participants skills in responding to the designed APR task, but it was not used to confirm or not the overall balance ability of the participants.

**Table 1:** **Numerical scoring system to quantify the APR's skill of participants**.

**Case**	**Score**
The participant maintained his hands and feet on its initial position	+2
The participant's hand(s) were displaced/unattached from its initial position	+1
The participant's feet were lifted from its initial position	−1
The participant was completely lost his balance and moved out of the platform	−2

## Results

### Number of utilized synergies

**Figure 5:**
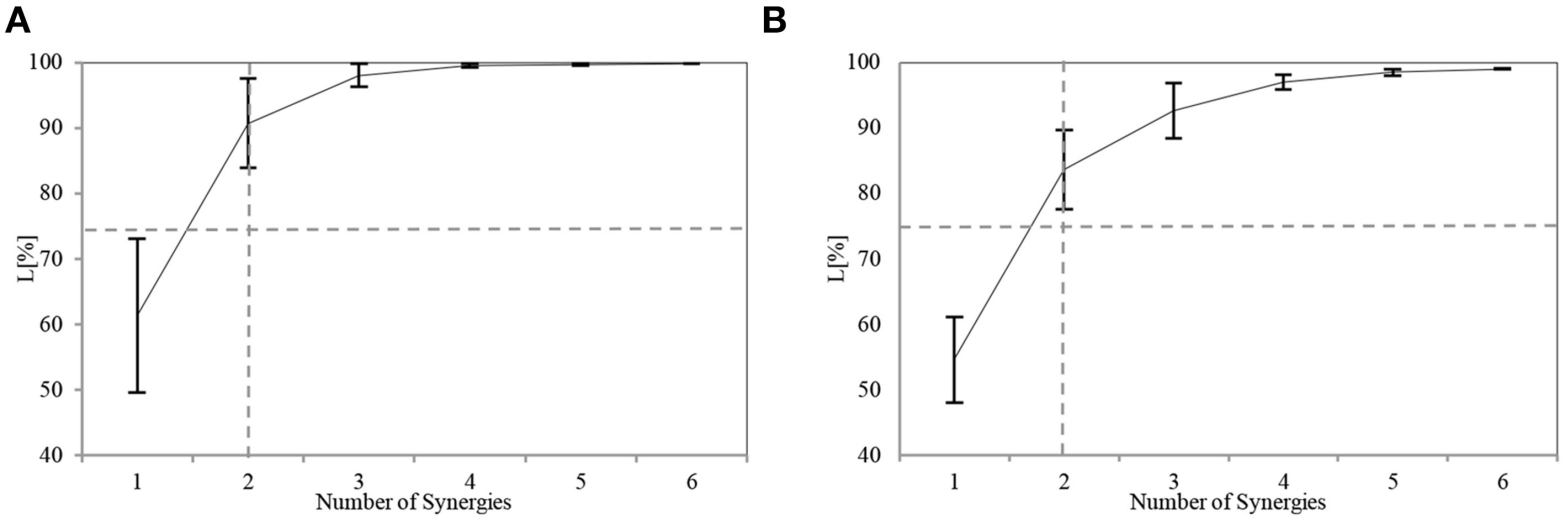
**Similarity *L* between the recorded and reconstructed (A) sensory data and (B) muscle activation patterns from all possible computed numbers of synergies (Equation 6)**. The plots show means and SD for each participant. The horizontal dashed line indicates the predefined threshold (75%), and the vertical dashed line indicates the selected number of utilized synergies.

All the participants successfully completed the assigned tasks, and their respective APR scores varied considerably. The number of utilized synergies *n* was the same across the participants. For sensory synergy, two synergies were enough to project the collected sensory data (Figure 5A). Similarly, two muscle synergies were enough to represent the measured muscle activations (Figure 5B). From these findings, the sensory or muscle synergies were analyzed on the assumption that two synergies were enough for each participant to complete the assigned task.

**Figure 6:**
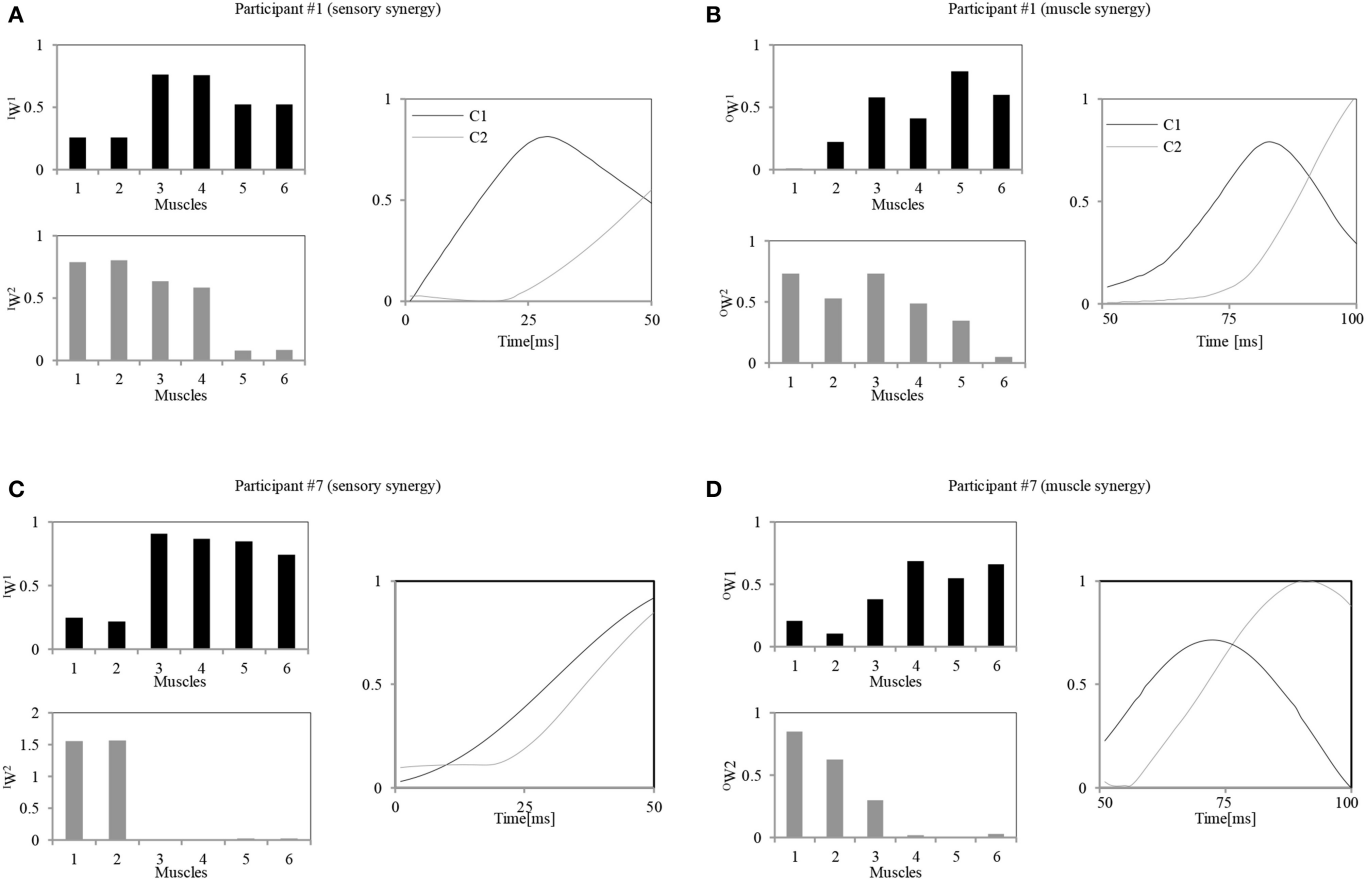
**Calculated sensory and muscle synergies for two representative participants. (A,C)** Sensory synergies for participant #1 / participant #7. **(B,D)** Muscle synergies for the participant #1 / participant #7. Muscle order: 1, FHL; 2, TA; 3, TFL; 4, GM; 5, RA; 6, ES.

Figure 6 shows an example of the resulting pair of synergies for two representative participants. Figures 6A,B show the sensory and muscle synergies computed from data for participant #1 (relatively good balance, score = 1.15), and Figures 6C,D show the sensory and muscle synergies computed from data for participant #7 (relatively poor balance, score = −0.9).

**Figure 7:**
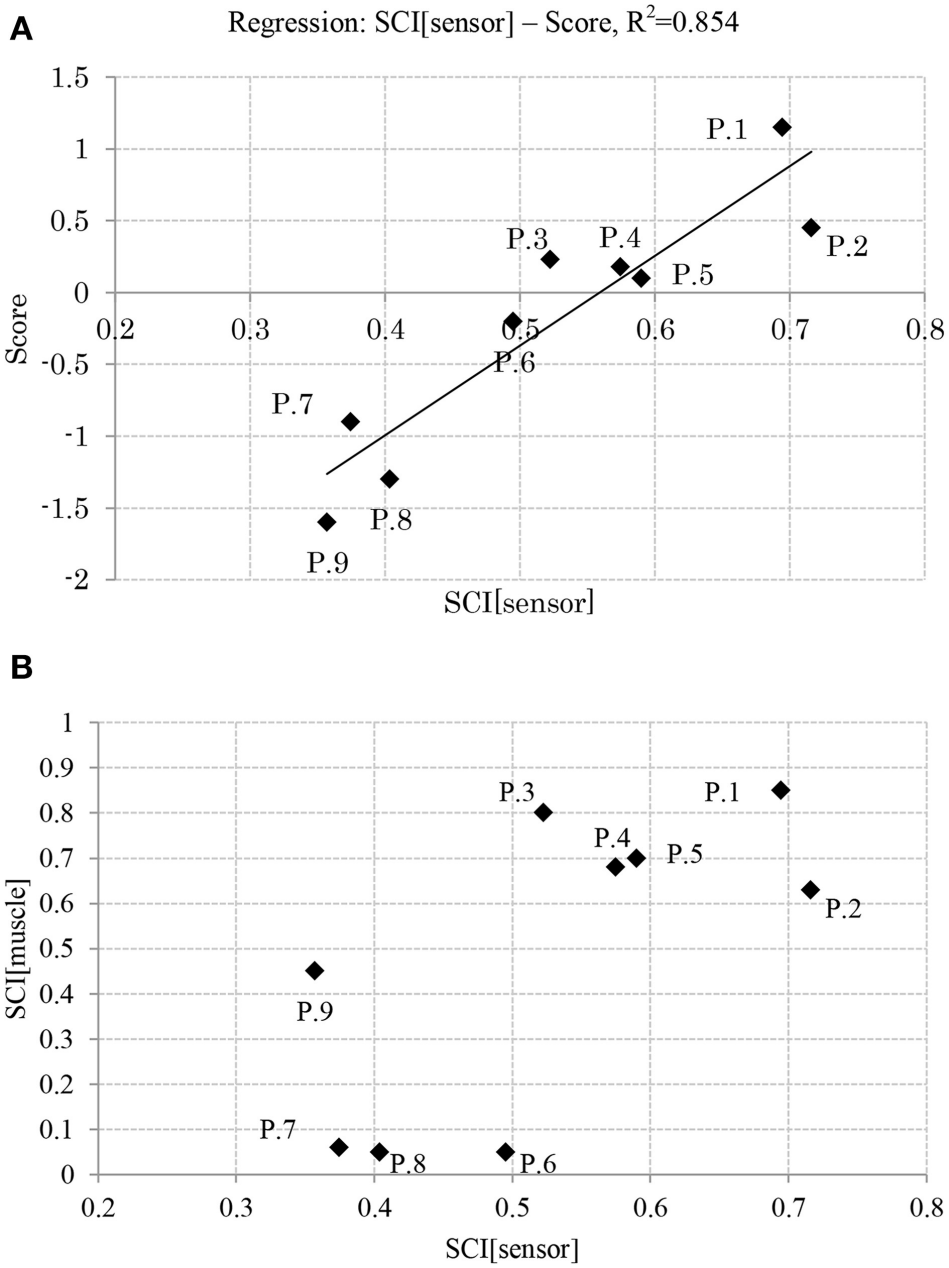
**(A)** Relation between balance skill level and sensory synergy size. **(B)** Relation between sensory synergy size and muscle synergy size. (P, Participant).

As seen in Figure 6, notably different strategies were adopted by each of the participants. These appear to represent their level of skill in responding to the disturbance. Participant #1, for instance, seems to have utilized two muscle synergies: one to control the lumbar region with the hip joints (*^O^W^(1)^*) and another to evoke the ankle and hip strategies (*^O^W^(2)^*). Similar strategies were also represented by the sensory synergies, where the ankle and the hip muscle length sensors were grouped together, and the hip and lumbar joint sensors were in another group. A correlation between the sensory and muscle synergy recruitment signals *^I^C* and *^O^C* was also observed. The control signal for precise posture control appeared with a delay of approximately 20 ms after the first signal. In contrast to these trends, participant #7 utilized an independent synergy for the ankle strategy alone (*^O^W^(2)^*), and another synergy to control the hip and the lumbar joints (*^O^W^(1)^*). Thus, the coordination between the utilization of these two muscle synergies seems to be weaker in participant #7 than participant #1. Also, the sensory and muscle synergy recruitment signals seem to show a poor match for this participant. The following two sections highlight the details of these characteristics and relate them to the balancing skills of the participants.

### Relation between APR's skill level and synergy size

Figure 7A shows the relation between the APR's skill level of the participants and their computed sensory synergy size, where the two appear to be directly proportional (the sensory synergy size is smaller for high-skill participants than for low-skill participants).

Figure 7B shows the relation between the sensory and muscle synergy sizes for all the participants, where it is clear that the sensory synergy seems to be consistent with the muscle synergy size. The correlations between sensory and muscle synergies are stronger when the synergy size is smaller.

### Relation between APR's skill and I/O similarity

**Figure 8:**
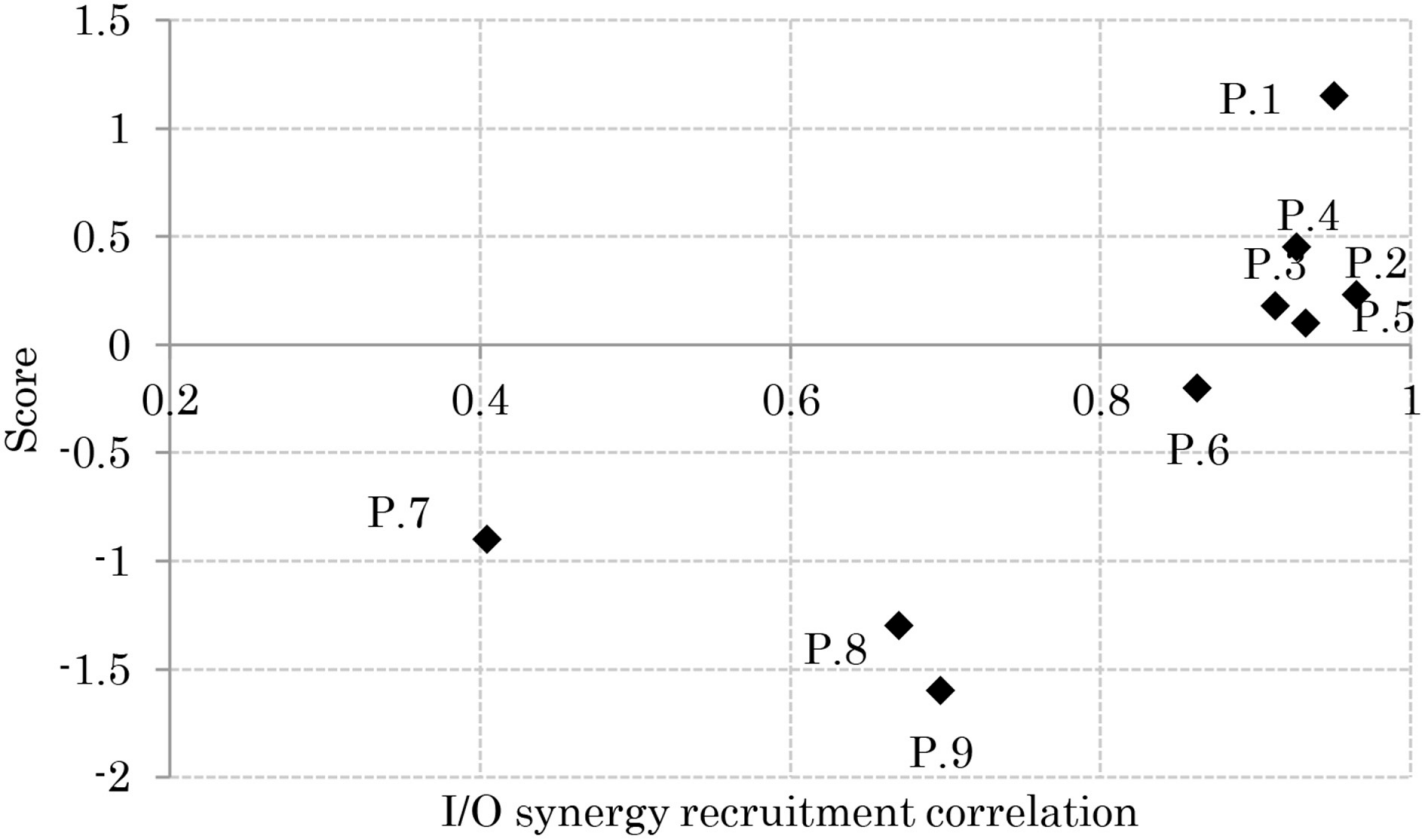
**Relation between balance skill level and sensory and muscle synergy recruitment signals**.

Figure 8 shows the relationship between the participants' scores and the correlation of their sensory and muscle synergies recruitments, *^I^C* and *^O^C*, respectively. From the figure, good performers show high correlation between the sensory/muscle synergies recruitments than bad performers. This high correlation could be the result of the smaller size of sensory and muscle synergies that facilitate mapping between environmental input and motor control.

## Discussion

### Relation between sensory and muscle synergies

This paper formulates a sensory synergy framework and emphasizes its advantage as a biologically plausible model that offers low-dimensional environmental input feedback that may improve on current approaches to neural prosthetic development. The main challenge in computing sensory synergy is to determine the relation between sensory and muscle synergies in a low-dimensional space. To that end, we adopted a simple task in which we used the changes on muscle length as a measure of the activation of proprioceptors over a period of 50 ms to estimate the sensory synergies. The period of 50 ms from the onset of muscle activities was considered in order to compute the muscle synergies. Only dominant muscles which have more influence in carrying out the motion were selected for synergies calculations. A posture control experiment with nine healthy participants was conducted to examine the relation between sensory and muscle synergies. The results suggest that the degree of coordination between the resulting sensory synergies (synergy size) can serve as an effective marker for characterizing to which extend the behavior is adapted to the environment.

Results reveal that participants with high APR scores showed well-tuned sensory synergies that project, in a smaller synergy size, a compendium of sensory data as feedback indicating the body posture. This smaller size suggests the existence of a sophisticated controller that simplifies and accelerates the transformation of the signal into a motor command, thus a correlation between the input *^I^C* and output *^O^C* was observed, and a control signal for precise posture recovery was emerged, Figures 6A,B. The smaller synergy size tends to show that joints are not controlled independently, thus guarantee a coordinated output movement, Figure 7B. In participants with weak scores, on the other hand, we observed a larger synergy size that suggests less trained controller which hardly was able to handle the introduced sensorimotor signaling, P7, P8, and P9, in Figures 7B, 8. The large synergy size that appeared in this group of participants, seems to cause passing larger amounts of unnecessary sensory information that may obstruct the formation of an optimal sensory signaling mapping to the desired motor control.

For future direction, we are planning to examine the contribution of other sensory modalities information, such as vision, center of pressure, etc., during a balance training phase that can be applied to the participants who only showed weak scores (Alnajjar et al., 2013b). We expect to observe an automatic converge of neural representation during the participants training that would increase the sensory weights for only those dominant sensors who mainly contributed to trigger muscle response and decrease the weights for those who were less efficient. This tuning of sensation weights could be depending on the task and the environment. The next stage of this study will be also targeting overcoming some of the limitations of this preliminary work. For instance, the subjective scoring system can be enhanced by abstracting it from the motion capture system. The time needed for the participant to recover his/her balance, or the degree of sway which is caused by the platform disturbance could be utilized to design a more robust scoring system.

From our initial results, we believe that sensory synergies are important to clarify low dimensional meaningful signals that simplify the work of the CNS when recruiting proper muscle synergies. It is also the key to determine the level of how much the body adapts to the surrounding environment. Designing prosthesis based upon the concept of sensory and muscle synergies can lead to make the controller simpler.

### Sensory synergy and the future of neuroprosthetics

A critical aspect of functional forearm prostheses is the ability to perform sensorimotor tasks. Mainstream powered forearm prostheses are controlled using surface EMG signals. The interface commonly uses EMG sensors to switch between different activation states of the prosthesis. With this control method, the user often experiences difficulty in learning how to control the prosthesis or how to generate an activation signal for a larger number of degrees of freedom and/or finer control of speed and force. Although research has been focusing on the motor control aspect, it is also very necessary to account for somatosensation, especially for proprioceptive and tactile modalities (Peerdeman et al., 2011).

Work on artificial hands indicates that a reduction in dimensionality can decrease the complexity of controlling prosthesis (Jerde et al., 2003; Katsiaris et al., 2012). The integration of tactile sense and proprioception is regarded as essential for implementing the ability to perceive environmental input (Rincon-Gonzalez et al., 2011). The identification of the sensory synergy onset may provide valuable cues that make it possible to extract the intent of the action, for example, the target of a reaching movement. Using sensory synergies is expected to allow for early recognition of the goal compared to when muscle synergies are used, as the latter is the result of modulation. This difference may be essential for implementing continuous and gentle movements in an activated system.

**Figure 9:**
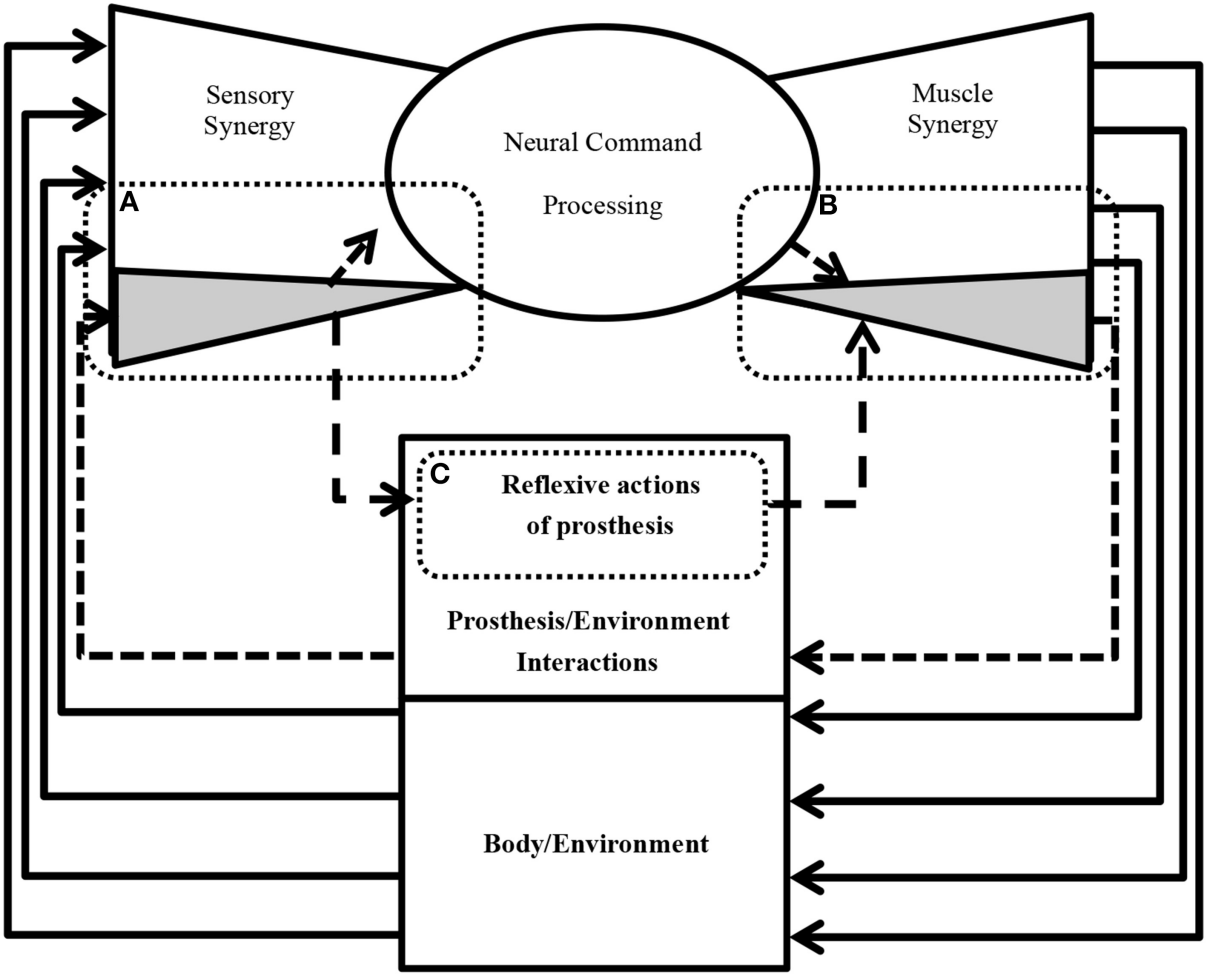
**A conseptual design of neural sensorimotor synergy system (future direction)**. **(A)** The acquired sensory signals and inferred artificial sensory synergies contribute to the sensory synergy. **(B)** The prosthesis controller computes motor commands based on the artificial sensory synergies. **(C)** The motor commands are recruited at the activators of the prosthesis in order to enable the wearer to control the motors. The motion of an artificial wrist joint adapts to the motion of the disarticulated arm in order to facilitate grasping and manipulation tasks.

Figure 9 shows an example of future practical applications of this study. The neural sensorimotor synergy system extends the system in Figure 1 by including prosthetic and exoskeletal artifacts. The dimensionality of the sensory stimulus is reduced through sensory synergy. A controller modulates sensory synergies to motor commands, and the modulation takes place in a space of reduced dimensionality compared to that of the input and output spaces. Motor commands are recruited at activators. In our ongoing research, we are applying this new principle of control to forearm prosthesis (Figure 9), and we are currently conducting clinical experiments involving the control of the forearm prostheses in accordance with the user's intention through the neural sensorimotor synergy system (Oyama et al., 2013; Iwatsuki et al., 2014). In short, from Figure 9, the dimensionality of the sensory stimulus to the prosthetic device is reduced by sensory synergy as part of the sensory system of the users, as illustrated in Figure 9A. The output from the sensory synergy is used as the input to both the CNS and an artificial controller. Compared with raw environmental inputs, the output from the sensory synergy should be easier to communicate to the CNS when sensory synergy is well defined. The control signals for the prosthetic device are created through motor synergy (Figure 9B). This synergy combines the signals from the CNS and the prosthesis controller and creates a higher-dimensional signal to control the prosthetic device. The prosthesis controller (Figure 9C) modulates the signal from the sensory synergy to the motor synergy. One of the roles of this prosthesis controller is the generation of reflexive motions to protect the users in case of unpredictable environmental changes.

## Author contributions

Conceived and designed the experiments: Fady Alnajjar, Shingo Shimoda. Performed the experiments: Fady Alnajjar, Shingo Shimoda. Analyzed the data: Fady Alnajjar, Matti Itkonen. Formed the equations: Fady Alnajjar, Shingo Shimoda. Wrote the paper: Fady Alnajjar, Matti Itkonen. Revised and discussed the paper: Fady Alnajjar, Matti Itkonen, Vincent Berenz, Maxime Tournier, Chikara Nagai, Shingo Shimoda.

### Conflict of interest statement

The authors declare that the research was conducted in the absence of any commercial or financial relationships that could be construed as a potential conflict of interest.
