# Supplementary material for: Sensory synergy as environmental input integration
Source: Front Neurosci. 2015 Jan 13;8:436. doi: 10.3389/fnins.2014.00436 (PMC4292368; doi:10.3389/fnins.2014.00436)
Supplement: Supplementary file 1 [file DataSheet1.pdf]

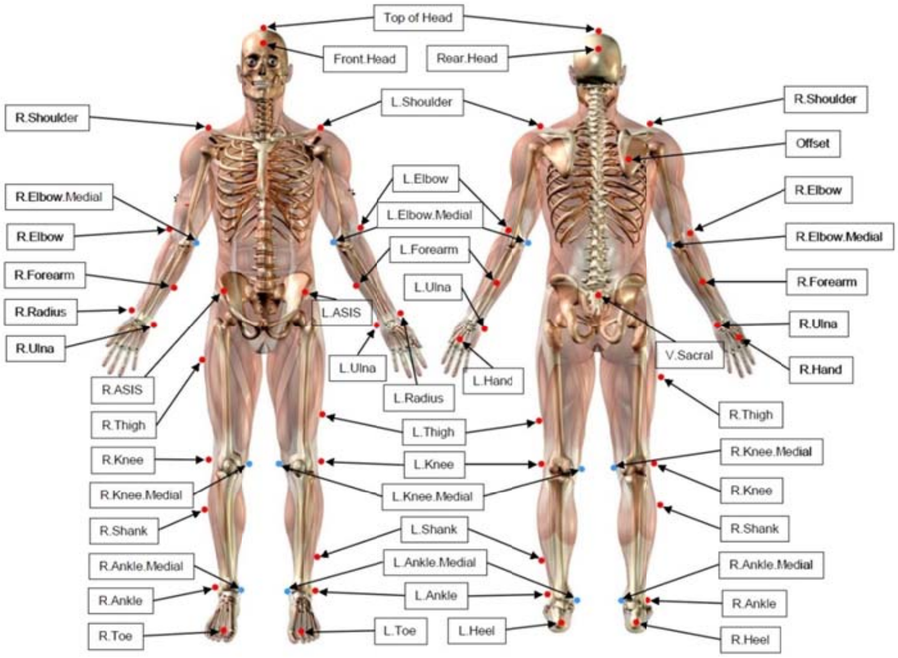

Supplementary Figure 1. Plug-in-Gait Marker Set.

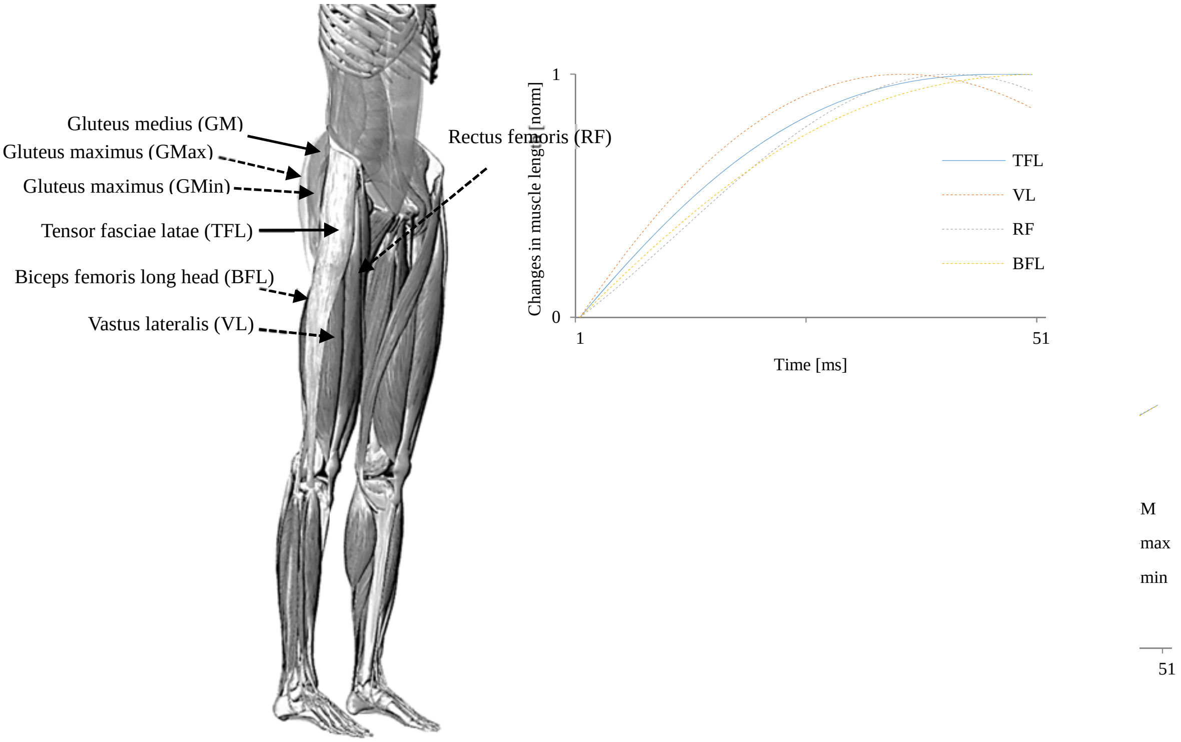

Supplementary Figure 2. An example of the changes on muscles lengths in the dominant and submissive muscles of P1. Dominant muscles (solid arrows), submissive muscles (dotted arrows).

In the offline analysis, we have noticed that the dominant muscles seem to reflect the common signal in its surrounding area. TFL, for instance, are a dominant muscle that represents the activities of the hip joint when responding to the lateral disturbances. Looking at the submissive muscles (RF, BFL, VL) that surround the TFL muscle, we can notice that the changes on muscle length of the submissive muscles did not add significant value to the final result, especially when considering the stability of their computed synergies. Submissive muscles could be well represented by the nearby dominant muscles.
